# Exploring patients and caregivers needs and experiences in oncological physiotherapy: a call for collaborative care

**DOI:** 10.1007/s00520-024-08782-y

**Published:** 2024-08-19

**Authors:** Almudena Medina-Rincón, Marta San Miguel-Pagola, Pablo Gargallo-Aguarón, Patricia Roldán-Pérez, Marina Francín-Gallego, Lorena Villa-García, Almudena Buesa-Estéllez

**Affiliations:** 1 Universidad San Jorge, Campus Universitario, Autov A23 Km 299, 50830 Zaragoza, Villanueva de Gállego Spain; 2 REFiT Aging Research Group, Parc Sanitari Pere Virgili and Vall d’Hebron Institute of Research (VHIR), Carrer d’esteve Terradas, 30 Gracia 08023, Barcelona, Spain; 3 Department of Public Health, Faculty of Nursing, Mental Health and Mother-Infant Nursing, University of Barcelona, L’Hospitalet de Llobregat, Barcelona, Spain

**Keywords:** Co-design, World Cafe, Cancer survivors, Humanization of care, Oncological physiotherapy, Rehabilitation

## Abstract

**Purpose:**

This study explores whether the full potential of physiotherapy is reaching cancer patients and their caregivers at all stages of the oncological process, aiming to identify gaps and opportunities for improving care.

**Methods:**

The World Cafe co-design methodology facilitated discussions among cancer patients and caregivers. This dynamic, inclusive, and engaging approach fostered diverse perspectives and deeper insights through collaborative and flexible discussions. Sessions were recorded, transcribed, and qualitatively analyzed.

**Results:**

Sixteen participants were involved (eight cancer survivors and eight caregivers). The mean age of cancer survivors was 63.8 years, while the average age of caregivers was 59.3 years. Breast cancer was the most prevalent diagnosis among patients, and most caregivers had lost their family members to cancer. Analysis revealed two primary themes: “feeling cared for” and “the role of physiotherapy in the oncological process.” Key findings highlight the need for more humanized healthcare, with professionals providing support through effective communication and empathy. Significant gaps were detected in both systematic referrals to physiotherapists and their integration into care teams. Testimonies highlighted the lack of knowledge about the full potential of physiotherapy in oncology, hindering access. There was also a demand for recognizing specialized oncological physiotherapists.

**Conclusions:**

These findings highlight significant gaps in physiotherapy care for cancer survivors and caregivers, including unmet needs due to the lack of information, resources, and effective communication. Future efforts should focus on increasing the visibility of physiotherapy, integrating specialized physiotherapists into oncology teams, and enhancing the emotional education of healthcare professionals to provide more humanized care.

**Supplementary Information:**

The online version contains supplementary material available at 10.1007/s00520-024-08782-y.

## Introduction

Nowadays, it is well-known that cancer has an ever-increasing incidence [1]. In Spain, the incidence in 2023 was 591 cases per 100,000 inhabitants [2], and it is estimated that in 2024 will reach 286,664 cases [3]. At the same time, the life expectancy of these patients is increasing due to earlier diagnosis, improvements in treatment, and a decrease in cardiovascular mortality [4]. The very sequelae of cancer and the aging population give rise to various disabilities that can be treated with physiotherapy, such as lymphedema, peripheral neuropathies, fatigue, poor physical condition, pain, or range-of-motion deficits [5]. Specifically, prescribing exercise to oncology patients helps to increase tolerance and decrease side effects before, during, and after treatment for all types of cancer [6], although its application is not yet widespread.

The Australian Society of Clinical Oncology has already recognized physiotherapists in its interdisciplinary teams, and the National Cancer Policy Forum in the United States published recommendations for better integration of rehabilitation services in cancer care, including physiotherapy [5]. The evidence supports the importance and necessity of physical therapy in the oncologic process [7, 8]. However, recognition and access to physiotherapy in the Spanish oncology population are still lacking [9, 10].

Both patients and caregivers perceive a lack of sufficient information about the resources available to them or even basic information that could empower them to actively participate during the rehabilitation process [11]. This lack of information on relevant aspects such as the types of adverse effects that may occur, how to detect and address them, or whom to consult with questions after medical discharge is often associated with higher levels of anxiety and depressive symptoms, negatively impacting their quality of life [12, 13].

Working collaboratively with patients and caregivers to explore and design how their care during cancer treatment is a necessary step to enhance their experience during the rehabilitation process. The collaborative design of strategies is known as co-design and is essential to ensure that care gaps are addressed, and services align with needs. Co-design refers to working in partnership (usually with end-users and providers) to design a service, program, or intervention aimed at improving the quality of care received by the service user [14]. There is emerging evidence suggesting that engaging with patients and caregivers as partners can lead to better care and a positive impact on users’ health [15]. This integrative methodology has been previously employed in various fields, such as in the realm of supportive care throughout different phases of the oncological process; however, to the authors’ knowledge, it is the first time it has been implemented in the field of oncological physiotherapy [16, 17].

This study aimed to explore whether the full potential of physiotherapy is reaching cancer patients and their caregivers at all stages of the oncological process, aiming to identify gaps and opportunities for improving care. The authors focused on tackling the widely recognized challenges encountered at various stages of cancer. By employing a co-creative approach, the goal was to enhance the physiotherapy services provided to this population.

## Methods

### Co-design framework

This study employed the World Café method, a participatory research technique within the qualitative methodology, to foster the collaborative exchange of ideas and creation of knowledge to address shared problems from the diverse perspectives of those involved [18–20]. This method facilitates capturing participant experiences and needs in a comfortable setting with smaller tables, refreshments, and rotating discussions, maintaining overall conversation cohesion [21]. This method was chosen because it allows the capture of participants’ lived experiences and desired services to address the needs of the population. Participants are not merely recipients of knowledge, but social actors involved in the knowledge process [19]. World Café has been used in a variety of contexts, including the development and evaluation of health services [22, 23], as well as in the improvement of care for the elderly [24, 25].

### Context and participants

#### Participant selection

The study was carried out at the Spanish Association Against Cancer (AECC) in Zaragoza, a non-profit association made up of patients, relatives, volunteers, and professionals related to oncology, which carries out its activity throughout Spain.

Eligible participants included adults diagnosed with cancer within the past 10 years, who are either currently undergoing treatment or have received any type of anticancer therapy, and who have sequelae from the disease and/or its treatment. Informal caregivers included adults who oversee (or have been) a family member or close person diagnosed with cancer, at any stage of the disease and not receiving remuneration for their caregiving. Exclusion criteria were physical or psychological conditions that precluded participation and a desire not to participate in the study.

#### Recruitment

All participants were recruited in September 2023 by the psychologist responsible for programs and services of the AECC and one of the members of the research team. The objectives of the project were explained to them through the information sheet to the participants and they were asked to sign the informed consent.

#### Sampling

The aim was to have between 6 and 12 participants per stakeholder group, with 13 participants invited to each group to ensure participation. Purposeful and in-depth sampling was conducted. To ensure heterogeneity of the sample, geographical diversity, the time in which the patients were concerning their disease, and the age and sex diversity of all the participants were considered.

### Data collection

#### Setting and data collection

The information was collected through World Café sessions, a survey that collected the sociodemographic variables of the participants and the field notes of the researchers. These sessions were held in person in AECC, away from the work areas and with little noise to guarantee privacy and avoid interruptions.

**Fig. 1:**
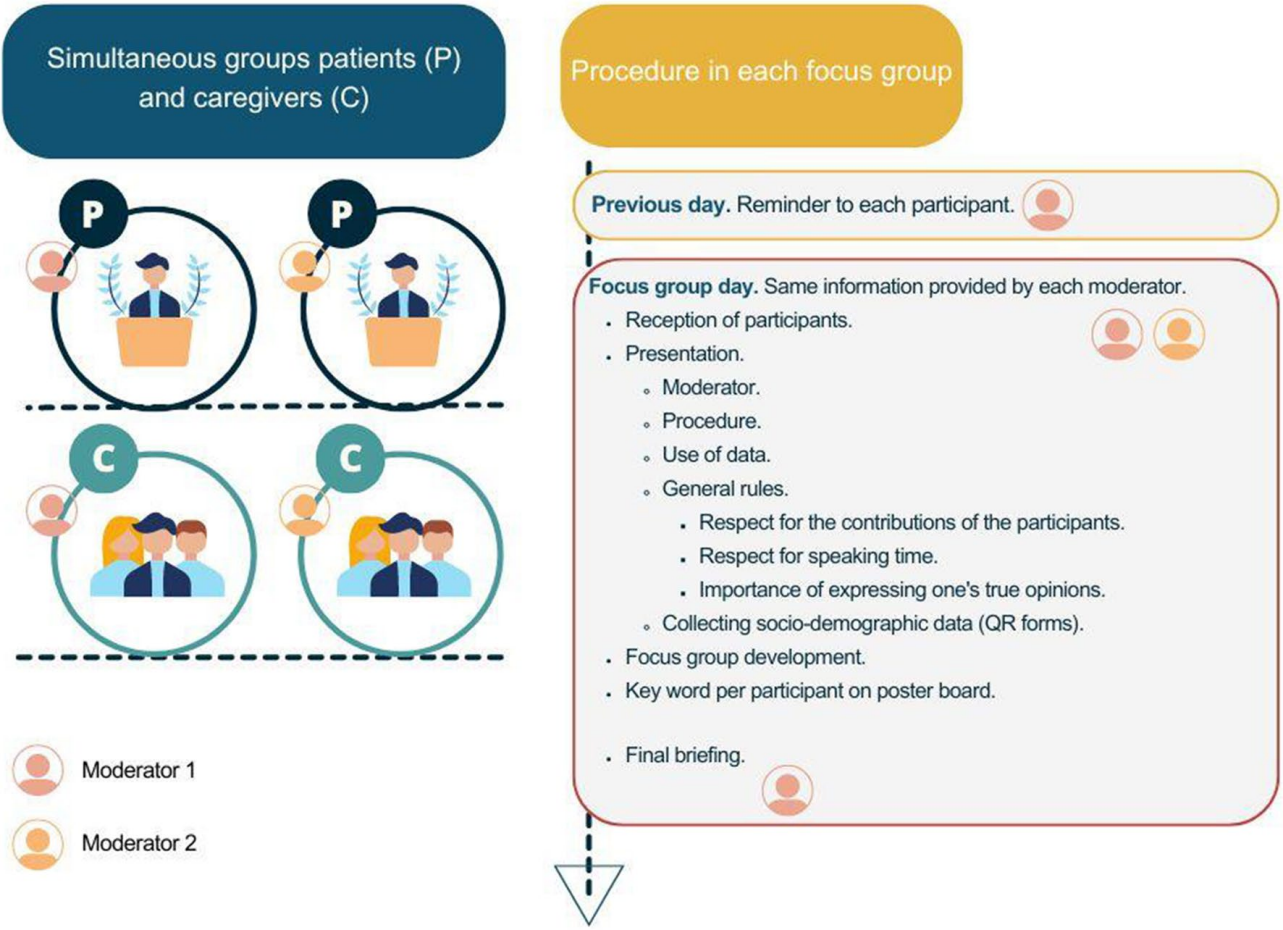
Procedure followed in the World Café sessions

Sessions were conducted between October and November 2023 following the procedure presented in Fig. 1. Each group lasted ~ 2 h. They were carried out by the research team, experienced in cancer patient care and qualitative methodology.

#### Research questions

During the sessions, a script of open-ended questions on categories of interest was followed (Table 1). This script was developed from the findings of a review of the research team’s experience. The questions were previously tested with five people to literature assess their form, sequence, and content.

**Table 1:** Question guide

**Perception of physical therapy**	1. What do you think physiotherapy can offer during the oncological process? 2. When do you think is the best time to receive physiotherapy treatment during the oncological process? Why?
**Description: previous experience with physiotherapy**	3. Can you describe any previous experience with physiotherapy programs or treatments linked to the oncological process?
**Person-centered care**	4. Did the physiotherapists who treated you consider your needs, habits, and preferences?
**Shared decision-making**	5. Do you think the physiotherapist involved you in the decision-making regarding treatment?
**Communication and information**	6. How do you rate the communication and information provided by the physiotherapist? 7. Do you think the physiotherapists who treat you communicate and coordinate with the rest of the professionals who treat you? What about your caregiver? (if you have)
**Accessibility**	8. How do you rate the accessibility of the physiotherapists who treat you? How do they do it? Is it easy for them to access them?
**Transitions**	9. How did you experience the transition of the doctor or professional who made the referral to the physiotherapy resource? (positives/negatives)
**Environment**	10. How would you describe the environment in which you interact with the physiotherapist? How does the environment affect you? 11. What differences do you see in people with cancer living in rural and urban areas? How does this influence your work? What about referral to the physiotherapy service?

### Transcripts

Audios from sessions were transcribed using the Amberscript software, ensuring fidelity to the original content without interpretation or modification. Participants’ identities were anonymized to protect confidentiality.

### Analysis

An inductive thematic analysis was used, consisting of the following steps: (i) read the transcriptions to get a general idea of identifying preliminary themes to organize the data; (ii) identify and classify the meaning units (MU) which are the smallest parts into which the text can be broken down, creating codes or descriptive categories; describing the grouping and distinguishing some codes from others; (iii) each grouping forms a unit of analysis, common meaning groups (CMG), based on the same characteristics; for further abstraction by grouping aspects that represent the thematic content; and (iv) synthesize the content of the condensation, creating a story based on the data reflecting content and meaning [26, 27]. A final consensus on the topics was reached after two triangulation sessions, and strategies to maintain methodological rigor were followed throughout the process.

### Methodological rigor

The Lincoln and Guba criteria were followed in research [28, 29]. The standards for reporting qualitative research (SRQR) and the consolidated criteria for reporting qualitative research (COREQ) standards ensured study validity [30, 31]. Table 2 shows all the techniques used to maintain the rigor.

**Table 2:** Methodological rigor strategies

Criteria	Description	Technique used	
Credibility	Confidence in the “truth “ of the findings	Research team triangulation	
		Member checking	
Transferability	Showing that the findings have applicablity in other contexts	Inspection of transcripts	
		Following a previously established correct protocol	
		Review the coding of specific parts	
		Communication between team members	
		Presentation of well-defined and described topics	
Dependability	Showing that the findings are consistent and could be repeated	Avoiding superficial coding	
		Avoiding the researcher’s interpretations	
		Adding negative cases	
		External auditor	
Confirmability	A degree of neutraility or the extent to which the findings of a study are shaped by the respondents and not researcher bias, motivation, or interest	Research herself/himself	Bracketing
		Participants	Avoid social desirability bias
			Member checking
		Research team	Triangulation and crystallization
			Peer debriefing
			Catalytic validity

*Ethical aspects.*

The study protocol has been prepared in accordance with the Helsinki Declaration and accepted by the Research Ethics Committee of the Autonomous Community of Aragon (CEICA), with the number C.I. PI23/306. All data have been stored in line with the general data protection regulation guidance.

## Results

### Sociodemographic and clinical data of the participants

A total of 16 participants were recruited (eight cancer survivors and eight caregivers). The mean age of people who had or were suffering from cancer was 63.8 years, and the average of caregivers was 59.3. In the case of patients, breast cancer was the most prevalent. Of the caregivers, except in two cases, their family member has already died. Also, two of them have been diagnosed with cancer. The descriptive characteristics of all participant groups can be found in Table 3.

**Table 3:** Participant characteristics

Cancer survivors				
ID	Gender	Age	Study levels	Cancer diagnosis
P_01	Male	64	Undergraduate education	Breast
P_02	Female	65	Non-university postsecondary education	Breast
P_03	Male	63	Undergraduate education	Gastric
P_04	Female	67	Undergraduate education	Breast
P_05	Male	66	Primary education	Kidney
P_06	Male	49	Undergraduate education	Prostate
P_07	Female	65	Non-university postsecondary education	Breast
P_08	Female	71	Upper secondary education	Breast
Caregivers				
ID	Gender	Age	Study levels	Relationship with the person with cancer
C_01	Male	64	Primary education	His wife died of breast cancer
C_02	Female	46	Non-university postsecondary education	Mother of a girl with pleural mesothelioma. She had uterine cancer
C_03	Male	72	Primary education	His wife died of pancreatic cancer. He was diagnosed with bladder cance
C_04	Female	57	Undergraduate education	Her husband has gastric cancer
C_05	Male	50	Undergraduate Education	His wife died of non-Hodgkin’s lymphoma
C_06	Male	69	Non-university postsecondary education	His wife died of lung cancer
C_07	Female	53	Undergraduate education	Her husband died of liver cancer
C_08	Male	63	Non-university postsecondary education	His wife died of pancreatic cancer

### Themes

Two main themes emerge from the thematic analysis: “feeling cared for” and “the role of physiotherapy in the oncological process”, which address aspects of the care patients and caregivers receive and the care they would like to receive based on a set of values, as well as the initial idea and generation of needs related to physiotherapy in the oncological specialty. The organization of each subtheme and its common meaning groups can be seen in Table 4. All narratives illustrating each subtheme and CMG can be found in the Supplementary Material.

**Table 4:** Themes and subthemes organization

Themes	Subthemes	Common meaning groups
Feeling cared for	Humanization	Vocation
		Empathy/misunderstanding (listening, eye contact, talking to the eyes)
		Ethics
		Communication
	Advice	Who does it
		Who doesn’t
	Access	Derivation—path of the public
		Resources—private (socio-economic impact)
		Rural/urban environment
	Specialization	Who knows what is doing
		Individualized treatment
The role of physiotherapy in the oncological process	Information: I didn’t know that physiotherapy is good for that/has potential	Preventive
		Treatment of sequelae
		Changing the myths
	Pillar in interdisciplinary teams	Absence
		Presence (need/vindication)
	Prior perception about physiotherapy	Negative emotions, pain, fear, embarrassment…
		Positive emotions

#### Theme 1: feeling cared for

The theme refers to the specific needs of participants for therapeutic accompaniment during the oncological process; they are calling for specialized healthcare professionals to provide support through effective communication and empathy.

##### Subtheme 1.1: humanization of care

Participants highlight fundamental aspects that health professionals who work with people with cancer should have. This first subtheme deals with aspects related to the vocation of professionals, empathy and misunderstanding, ethics, and communication. All of these are considered essential aspects of the health profession.

Vocation refers to the good treatment received by the professional, who sometimes skips the “established protocol” to offer the best care:

“She lived it, she liked it, she loved it and then you could see her as a kind, generous woman. So, to you who are in those moments, who are first of all scared, who do not know what is going to happen. Well, man, to be treated a certain way is fabulous... For me, the most important thing was the treatment.” (P_06).

Many patients and caregivers describe a lack of empathy from healthcare professionals. Empathy may facilitate the process of addressing the challenges encountered during the oncologic process, so participants emphasize the imperative need to change:

“I need, I mean, I miss more empathy, that is, that they put themselves more in your place.” (P_07)

When the participants talk about ethics, they emphasize that healthcare workers are guided more by hospital protocols than by work ethics.

Regarding communication, several situations can be identified: caregivers who notice that health workers provide different explanations each time or limit the information they communicate; communication difficulties regarding gender; and the need for both patients and caregivers to get together and tell each other about their experiences.

“I’m going to accompany him and find out a little, because then everyone tells you what they want.” (C_04).

##### Subtheme 1.2: advice

Counseling was considered important and there was variability in the forms and professionals involved. In it, the participants, but especially the caregivers, explain from whom they have received support and advice and from whom they have not.

Participants expressed discrepancies in the level of engagement of different healthcare professionals. While oncologists tended to provide comprehensive information and guidance, some surgeons seemed to focus solely on eradicating the cancer, neglecting to address potential adverse effects such as lymphedema. One participant highlighted the supportive role of the oncologist, comparing him to a “guardian angel”, while another lamented the lack of advice regarding the treatment of lymphedema.

In general, participants expressed a lack of professional advice. Some participants mentioned that they had sought information.

“Either you make a living, or no one helps you.” (C_05) or sought advice from cancer charities and found this helpful “My wife didn’t eat either. Everything gave her… At the Cancer Association, the nutritionist was the first person who gave me some guidelines for food.” (P_03).

Caregiver 03 talks about how he was unable to learn how to clean and move his wife by the auxiliary nursing staff, which would have influenced a better quality of life.

“There’s one thing that I’m missing a lot, and that’s that every time they came to either make the bed or change it, or this or that, the only thing they just did was throw you out of the room.” “So sometimes I said, ‘Why don’t you let me see how you pick it up, how you move it, how you change the sheet?” (C_03).

Especially about bed mobilizations and transfers, this lack of advice leads caregivers to feel “on edge”, and burnt out, trying to take care of their family members in the best possible way, but without receiving help from health professionals.

##### Subtheme 1.3: access

In it, the participants recount the different routes and the difficulties they encounter to be able to access some services, necessary in the oncological process.

In the referral process, for example, to physiotherapy treatment, there is a general feeling of having to make a living because the care provided by public health has an expiration date.

“Well, this path of the public has had to end because no one tells me anything, because the information and then I go on my own.” (P_03).

Most participants identify inequalities in access to physical therapy services determined by social class and geographic area of residence.

*“If this happens to you in a village, you can die now.” (P_01).*

##### Subtheme 1.4: specialization

This relates to the general idea that physiotherapy in the oncological process is very specific, especially when lymphedema is present. This specialized training is crucial to ensure adequate care and results, as shown by the experience of P_01.

“Well, for those 15 days the headline was fine, but she went on holiday. A girl from Granada came, a young girl, she was very nice, she was very worried, but well, she didn’t have a clue. I had to keep telling her what I had to do. Look, now put the sleeves on and do this, ... and now bandage me. So of course, the first day he bandaged me terribly, my hand got...!” (P_01).

#### Theme 2: the role of physiotherapy in the oncological process

Although some patients and caregivers had a previous perception of physiotherapy, they were surprised to understand that it is a key part of the treatment team of people with cancer, claiming its necessity in the whole oncological process.

##### Subtheme 2.1: “info: I didn’t know that physiotherapy is good for this/has that potential”

Both patients and caregivers expressed their lack of knowledge about all the aspects in which physiotherapy can intervene, both in health promotion and prevention and in the treatment of sequelae.

As a preventive treatment, even if they do not name it directly, participants become aware of the relationship that movement has with better health.

“Physical therapy, I think, is good even if it’s just for blood circulation, something as simple as that.” (C_03).

While physiotherapy is often sought for pain relief, it holds particular significance for cancer patients who have had lymphatic system involvement. These patients emphasize the lasting impact that rehabilitation has had on their lives.

As one participant stated: “Moving, giving yourself a massage makes you more alive. I think it’s very important.” (P_03)*.* Additionally, cancer has taught them that physiotherapy encompasses a wide range of treatments, including cardiorespiratory and neuropediatric care.

“Physiotherapy also has to do not only with muscle physiotherapy but also with pulmonary, cardiorespiratory.” (P_03).

Although physiotherapy has a long history, participants consider that it is something emerging. However, this perception is evolving it is no longer just about giving massages, it is about health education and therapeutic exercise.

“If they help you, you can do a little healthier living, they give you guidelines for those guided walks, that I don’t think physiotherapy is just giving massages. For me, physiotherapy should be more things.” (P_03).

##### Subtheme 2.2: interdisciplinary oncology team

Participants identify that physiotherapy has benefits and that it should be a mainstay in the interdisciplinary oncology teams to ensure better care and quality of life. Many participants expressed the urgent need to include physical therapy.

“From minute zero, they should be part of it. The team that treats you is multidisciplinary, but it includes oncologists, surgeons, even a psychologist. But where’s the physiotherapy?” (P_02).

##### Subtheme 2.3: previous perceptions regarding physiotherapy

Most participants had previous experience of physiotherapy, in some cases for other conditions. Some participants described negative previous emotions, which are those related to fear, pain, and shame (which have to do mainly with gender).

“My wife just didn’t want to because I don’t know, ... She has also admitted that she was very embarrassed to undress.” (C_01).

As positive emotions, the participants have found in physiotherapy the solution and response to some of their ailments.

“So, opiates were a solution to take away the pain, but not to take away the problem that the physio later discovered, which is that he had some edema, some broken bones and such.” (C_07).

## Discussion

To the authors’ knowledge, this is the first study to utilize the World Café methodology to explore the experiences and real needs of individuals with cancer and their caregivers regarding oncological physiotherapy. This study revealed a widespread lack of visibility about physiotherapy’s role in cancer treatment and highlighted the need for enhanced humanized guidance and support for patients across all disease stages.

While there has been growing interest in recent years in improving physiotherapy intervention by exploring the perceptions and experiences of individuals with cancer [32–34], the World Café methodology has, until now, only been employed in studies with participants from other profiles. Previous studies have used this methodology to explore the barriers and facilitators of access to palliative care, including physiotherapy, for individuals with incurable cancer, but these studies included healthcare professionals as participants rather than patients or caregivers and focused on a single phase of the oncological process, palliative care [35].

Although not specifically focused on oncological physiotherapy, it is interesting to compare those co-design studies that have included, as in this study, the perspectives of patients and caregivers on different rehabilitation processes [36–38]. In these studies, one of the recurring themes in the results is the unmet needs of individuals with cancer and their caregivers [39]. Many people with cancer do not express their needs at the beginning of rehabilitation due to various factors such as communication with medical staff, lack of empathy, or time constraints during medical consultations [40]. This coincides with several testimonies collected in this study, which point out the absence of empathy from some professionals in addressing issues related to their rehabilitation needs, or the conflicting information they receive from different professionals. Limited consultation times and the stress of receiving prognostic news can prevent patients from fully expressing their needs. For this reason, promoting effective communication can reduce anxiety and improve treatment satisfaction by fostering trust and patient involvement in decision-making [41, 42]. Finding quality spaces where a member of the care team can address patients’ concerns and provide health education could be part of the solution. Upon reviewing the scientific literature, it becomes evident that the dehumanization of care is a common issue faced by patients within the healthcare system [43]. Enhancing the emotional education of healthcare professionals may better equip them to provide care in a more humanized and compassionate manner.

The lack of information throughout the disease process is one of the subthemes expressed by participants in this study, and as described in other studies, this lack of information tends to generate anxiety/distress and a sense of insecurity, potentially diminishing their perception of health-related quality of life [12, 13]. Gathering patient concerns to create expert-verified informational material is essential, with new technologies and artificial intelligence playing a crucial role in dissemination [44].

Another problem reported by participants is the difficulty of accessing these services, supported by other studies indicating insufficient and unsystematic referrals [45, 46]. This situation is particularly prevalent in types of incurable cancer [47]. In the case of breast cancer, the most prevalent type of cancer, several studies reflect a much lower referral rate than necessary [48]. Additionally, social inequalities further condition access to rehabilitation services [49]. Highlight the potential of physiotherapy in the different phases of the oncological process could improve access to it. Tools such as telerehabilitation could help reduce inequalities [50].

Besides the physical and psychosocial consequences of the disease and its treatments, there is a significant economic burden associated with cancer known as financial toxicity, which can affect a significant number of people with cancer and their families, especially those in more vulnerable situations [51]. Participants noted the economic impact of limited access to public physiotherapy, leading to inequality. Yet, support from the AECC team (nutrition, physiotherapy, and psychology) was praised. This illustrates how a non-profit organization has helped meet the rehabilitation needs of people with cancer, aligning with recent studies such as the one conducted in Canada, emphasizing the importance of bringing non-profit organizations closer to the cancer community to improve support and meet their needs [52].

Regarding the second theme, “the role of physiotherapy in the oncological process,” this study’s results indicate the need for physiotherapists to specialize in oncology. Unpleasant experiences with physiotherapists unfamiliar with cancer were highlighted. This aligns with other studies where healthcare professionals discussed the same topic [53, 54]. The lack of information about addressing cancer sequelae in physiotherapy is another subtheme mentioned by people with cancer and their caregivers. Current evidence supports the beneficial effects of physiotherapy for sequelae such as lymphedema or pelvic floor dysfunctions [8], but there is a considerable gap between research and clinical practice [55]. Creating a network of collaboration between research centers and non-profit organizations more connected to society could help mitigate this problem.

Another subtheme addressed is the cognitive barrier that some men present in expressing rehabilitation needs, especially when it comes to issues related to incontinence or sexual health. This was already manifested in the study by Neris and colleagues, where they highlight the social, physical, and emotional repercussions experienced by men with urological cancer, who perceive their masculinity as threatened [56]. Considering that the global cancer incidence is slightly higher in men, it is crucial to dismantle gender barriers and create safe spaces of respect and trust.

The main strengths of the study include assessing the unmet needs and previous experiences concerning oncological physiotherapy by using an innovative co-design approach that considers patients and caregivers with experience in different stages of cancer. A few limitations should also be mentioned. Firstly, participants of the study were only cancer patients and their caregivers, but other stakeholders such as health professionals, managers, or others might have been included. In addition, only unpaid carers were included, so professional carers may have other needs or experiences that could also be interesting to explore. Future research by this group is scheduled to address these aspects. Secondly, most of the cancer patients included in the Cancer Survivors group had a breast cancer diagnosis. Despite being one of the most prevalent cancer types and therefore being representative somehow, the inclusion of survivors of different cancer types would have been even more enriching.

Our findings reveal significant gaps in comprehensive physiotherapy care for cancer survivors and caregivers, including unmet rehabilitation needs due to a lack of information, specialty care, resources, empathy, and communication with healthcare professionals. To address these issues, future efforts should prioritize increasing the visibility and awareness of physiotherapy, integrating specialized physiotherapists into interdisciplinary oncology teams, and enhancing effective communication and the emotional education of healthcare professionals. These steps are crucial to ensure better access and support for patients and caregivers throughout the cancer journey.

## Supplementary Information

Below is the link to the electronic supplementary material.

Supplementary file1 (DOCX 29 KB)

## Data Availability

The data underlying this study are available in the article and its online supplementary materials, except some words that have not been included due to the risk of re-identification.

## References

[1] Bray F, Laversanne M, Sung H et al (2024) Global cancer statistics 2022: GLOBOCAN estimates of incidence and mortality worldwide for 36 cancers in 185 countries. CA Cancer J Clin 74:229–263. 10.3322/caac.21834 38572751 10.3322/caac.21834

[2] Asociación Española Contra el Cáncer (2023) Dimensiones del cáncer. Asociación Española Contra el Cáncer. https://observatorio.contraelcancer.es/explora/dimensiones-del-cancer. Accessed 17 July 2024

[3] Sociedad Española de Oncología Médica (2024) Las cifras del cáncer en España 2024. Sociedad Española de Oncología Médica. https://seom.org/images/publicaciones/informes-seom-de-evaluacion-de-farmacos/LAS_CIFRAS_2024.pdf. Accessed 17 July 2024

[4] Costa AR, Alves L, Lunet N (2020) Healthcare services and medication use among cancer survivors and their partners: a cross-sectional analysis of 16 European countries. J Cancer Surviv 14:720–730. 10.1007/s11764-020-00886-8 32594450 10.1007/s11764-020-00886-8

[5] Stuiver MM, Stout NL, Dennett AM et al (2019) An international perspective on integrating physiotherapists in oncology care. J Physiother 65:186–188. 10.1016/j.jphys.2019.07.004 31477498 10.1016/j.jphys.2019.07.004

[6] McNeely M, Dolgoy N, Onazi M, Suderman K (2016) The interdisciplinary rehabilitation care team and the role of physical therapy in survivor exercise. Clin J Oncol Nurs 20:S8–S16. 10.1188/16.CJON.S2.8-16 27857275 10.1188/16.CJON.S2.8-16

[7] Stout NL, Harrington SE, Perry A, Alappattu MJ, Pfab V, Stewart B, Manes MR (2023) Implementation of a cancer rehabilitation navigation program: a qualitative analysis of implementation determinants and strategies. J Cancer Surviv 26:1–4. 10.1007/s11764-023-01374-5 10.1007/s11764-023-01374-5 37099228

[8] Stout NL, Santa Mina D, Lyons KD et al (2021) A systematic review of rehabilitation and exercise recommendations in oncology guidelines. CA cancer J Clin 71:149–175. 10.3322/caac.21639 33107982 10.3322/caac.21639 PMC7988887

[9] Galiano-Castillo N, Postigo-Martin P, Cantarero-Villanueva I (2020) The role of physical therapists in oncology: the great unknown. Phys Ther Rev 25:235–237. 10.1080/10833196.2020.1804783

[10] Herrero López B, Cardeña-Gutiérrez A, Godoy Ortiz A, et al (2024) Exercise in cancer patients: assistance levels and referral pathways—a position statement from the Spanish Society of Medical Oncology. Clin Transl Oncol. 10.1007/s12094-024-03546-w 10.1007/s12094-024-03546-w PMC11735567 38909323

[11] Yang Y, Chen X, Pan X et al (2023) The unmet needs of patients in the early rehabilitation stage after lung cancer surgery: a qualitative study based on Maslow’s hierarchy of needs theory. Support Care Cancer 31:1–12. 10.1007/s00520-023-08129-z 10.1007/s00520-023-08129-z 37934256

[12] Hansen DG, Larsen PV, Holm LV et al (2013) Association between unmet needs and quality of life of cancer patients: a population-based study. Acta Oncol (Madr) 52:391–399. 10.3109/0284186X.2012.742204 10.3109/0284186X.2012.742204 23244672

[13] Møller JK, Jespersen E (2019) Associations between perceived information needs and anxiety/depressive symptoms among cancer caregivers: a cross-sectional study. J Psychosoc Oncol 0:1–17. 10.1080/07347332.2019.1664699 10.1080/07347332.2019.1664699 31535929

[14] Castro EM, Malfait S, Van Regenmortel T et al (2018) Co-design for implementing patient participation in hospital services: a discussion paper. Patient Educ Couns 101:1302–1305. 10.1016/j.pec.2018.03.019 29602511 10.1016/j.pec.2018.03.019

[15] Bombard Y, Baker GR, Orlando E et al (2018) Engaging patients to improve quality of care: a systematic review. Implement Sci 13:98. 10.1186/s13012-018-0784-z 30045735 10.1186/s13012-018-0784-z PMC6060529

[16] van Strien-Knippenberg IS, Boshuizen MCS, Determann D et al (2022) Cocreation with Dutch patients of decision-relevant information to support shared decision-making about adjuvant treatment in breast cancer care. Heal Expect 25:1664–1677. 10.1111/hex.13510 10.1111/hex.13510 PMC9327829 35579109

[17] Grant AR, Koczwara B, Morris JN et al (2021) What do cancer survivors and their health care providers want from a healthy living program? Results from the first round of a co-design project. Support Care Cancer 29:4847–4858. 10.1007/s00520-021-06019-w 33544245 10.1007/s00520-021-06019-w

[18] Recchia V, Dodaro A, De Marco E, Zizza A (2022) A critical look to community wisdom: applying the World Café method to health promotion and prevention. Int J Health Plann Manage 37:220–242. 10.1002/hpm.3594 36411997 10.1002/hpm.3594

[19] Fouché C, Light G (2011) An invitation to dialogue. Qual Soc Work 10:28–48. 10.1177/1473325010376016

[20] Brown J (2005) The World Café: shaping our futures through conversations that matter. Berrett-Koehler Publishers, San Francisco

[21] Löhr K, Weinhardt M, Sieber S (2020) The “World Café” as a participatory method for collecting qualitative data. Int J Qual Methods 19:160940692091697. 10.1177/1609406920916976

[22] MacFarlane A, Galvin R, O’Sullivan M et al (2016) Participatory methods for research prioritization in primary care: an analysis of the World Café approach in Ireland and the USA. Fam Pract 34:cmw104. 10.1093/fampra/cmw104 10.1093/fampra/cmw104 PMC6080563 27677298

[23] Broom M, Brady B, Kecskes Z, Kildea S (2013) World Café methodology engages stakeholders in designing a neonatal intensive care unit. J Neonatal Nurs 19:253–258. 10.1016/j.jnn.2012.12.002

[24] Yankeelov PA, Faul AC, D’Ambrosio JG et al (2019) World Cafés create healthier communities for rural, older adults living with diabetes. Health Promot Pract 20:223–230. 10.1177/1524839918760558 29557175 10.1177/1524839918760558

[25] Khong L, Bulsara C, Hill KD, Hill AM (2017) How older adults would like falls prevention information delivered: fresh insights from a World Café forum. Ageing Soc 37:1179–1196. 10.1017/S0144686X16000192

[26] Malterud K (2012) Systematic text condensation: a strategy for qualitative analysis. Scand J Public Health 40:795–805. 10.1177/1403494812465030 23221918 10.1177/1403494812465030

[27] Hashimov E (2015) Qualitative data analysis: a methods sourcebook and the coding manual for qualitative researchers. Tech Commun Q 24:109–112. 10.1080/10572252.2015.975966

[28] Roldán-Pérez P, San Miguel-Pagola M, Doménech-García V et al (2023) Identification of the needs of children with neurodisability and their families at different stages of development: a qualitative study protocol. PLoS ONE 18:e0291148. 10.1371/journal.pone.0291148 37682853 10.1371/journal.pone.0291148 PMC10490905

[29] Lincoln YS, Guba EG (1985) Naturalistic inquiry. SAGE Publications, Newbury Park

[30] O’Brien BC, Harris IB, Beckman TJ et al (2014) Standards for reporting qualitative research: a synthesis of recommendations. Acad Med 89:1245–1251. 10.1097/ACM.0000000000000388 24979285 10.1097/ACM.0000000000000388

[31] Tong A, Sainsbury P, Craig J (2007) Consolidated criteria for reporting qualitative research (COREQ): a 32-item checklist for interviews and focus groups. Int J Qual Heal Care 19:349–357. 10.1093/intqhc/mzm042 10.1093/intqhc/mzm042 17872937

[32] Bennett AE, O’Neill L, Connolly D et al (2018) Patient experiences of a physiotherapy-led multidisciplinary rehabilitative intervention after successful treatment for oesophago-gastric cancer. Support Care Cancer 26:2615–2623. 10.1007/s00520-018-4112-6 29455302 10.1007/s00520-018-4112-6

[33] Fordham B, Smith TO, Lamb S et al (2022) Patient and physiotherapist perceptions of the getting recovery right after neck dissection (GRRAND) rehabilitation intervention: a qualitative interview study embedded within a feasibility trial. BMJ Open 12:1–11. 10.1136/bmjopen-2022-064269 10.1136/bmjopen-2022-064269 PMC9664296 36375975

[34] Land J, Hackett J, Sidhu G et al (2022) Myeloma patients’ experiences of a supervised physical activity programme: a qualitative study. Support Care Cancer 30:6273–6286. 10.1007/s00520-022-07062-x 35467117 10.1007/s00520-022-07062-x PMC9035778

[35] Mendieta CV, de Vries E, Gomez-Neva ME et al (2023) Barriers and facilitators to palliative care for patients with non-curable cancer in Colombia: perspectives of allied health and social care professionals. BMC Palliat Care 22:1–10. 10.1186/s12904-023-01267-5 37798738 10.1186/s12904-023-01267-5 PMC10557296

[36] Villa-García L, Davey V, Peréz LM, et al (2023) Co-designing implementation strategies to promote remote physical activity programs in frail older community-dwellers. Front Public Heal 11:. 10.3389/fpubh.2023.1062843 10.3389/fpubh.2023.1062843 PMC10028273 36960372

[37] Yang CL, Labbé D, Sakakibara BM et al (2022) World Café- a community conversation: a Canadian perspective on stroke survivors needs for community integration. Top Stroke Rehabil 29:392–400. 10.1080/10749357.2021.1928839 34057404 10.1080/10749357.2021.1928839

[38] Hartford W, Lear S, Nimmon L (2019) Stroke survivors’ experiences of team support along their recovery continuum. BMC Health Serv Res 19:1–12. 10.1186/s12913-019-4533-z 31638959 10.1186/s12913-019-4533-z PMC6805495

[39] Wang T, Molassiotis A, Pui B et al (2018) Unmet care needs of advanced cancer patients and their informal caregivers: a systematic review. BMC Palliat Care 17:1–29. 10.1186/s12904-018-0346-9 10.1186/s12904-018-0346-9 PMC6057056 30037346

[40] Heß V, Meng K, Schulte T et al (2020) Prevalence and predictors of cancer patients’ unexpressed needs in the admission interview of inpatient rehabilitation. Psychooncology 29:1549–1556. 10.1002/pon.5450 32602575 10.1002/pon.5450

[41] Park S, Kim H-K, Lee M (2023) An analytic hierarchy process analysis for reinforcing doctor–patient communication. BMC Prim Care 24:24. 10.1186/s12875-023-01972-3 36670353 10.1186/s12875-023-01972-3 PMC9860231

[42] Barry MJ, Edgman-Levitan S (2012) Shared decision making — the pinnacle of patient-centered care. N Engl J Med 366:780–781. 10.1056/NEJMp1109283 22375967 10.1056/NEJMp1109283

[43] Busch IM, Moretti F, Travaini G et al (2019) Humanization of care: key elements identified by patients, caregivers, and healthcare providers. A systematic review. Patient - Patient-Centered Outcomes Res 12:461–474. 10.1007/s40271-019-00370-1 10.1007/s40271-019-00370-1 31203515

[44] Li Y, Gao W, Luan Z et al (2023) The impact of chat generative pre-trained transformer (ChatGPT) on oncology: application, expectations, and future prospects. Cureus 15:1–8. 10.7759/cureus.48670 10.7759/cureus.48670 PMC10714025 38090410

[45] Cho S, Chung SH, Kang M et al (2021) Underutilisation of physical rehabilitation therapy by cancer patients in Korea: a population-based study of 958,928 Korean cancer patients. J Korean Med Sci 36:1–12. 10.3346/JKMS.2021.36.E304 10.3346/jkms.2021.36.e304 PMC8629717 34845872

[46] Ross L, Petersen MA, Johnsen AT et al (2012) Are different groups of cancer patients offered rehabilitation to the same extent? A report from the population-based study “the cancer patient’s world.” Support care cancer 20:1089–1100. 10.1007/s00520-011-1189-6 21597939 10.1007/s00520-011-1189-6

[47] Loughran K, Rice S, Robinson L (2019) Living with incurable cancer: what are the rehabilitation needs in a palliative setting? Disabil Rehabil 41:770–778. 10.1080/09638288.2017.1408709 29185362 10.1080/09638288.2017.1408709

[48] Falcicchio C, Di Lallo D, Fabi A et al (2021) Use of rehabilitation pathways in women with breast cancer in the first 12 months of the disease: a retrospective study. BMC Cancer 21:311. 10.1186/s12885-021-07927-0 33761916 10.1186/s12885-021-07927-0 PMC7993006

[49] Cogollos-de-la-Peña R, Álvarez-Vargas A, Domínguez-Navarro F et al (2024) Social inequalities in the use of physiotherapy in women diagnosed with breast cancer in Barcelona: DAMA cohort. Breast Cancer Res Treat 204:377–387. 10.1007/s10549-023-07191-9 38155271 10.1007/s10549-023-07191-9 PMC10948522

[50] Gupta E, Mitchell CH, Ngo-Huang A et al (2023) Addressing social determinants of health to reduce disparities among individuals with cancer: insights for rehabilitation professionals. Curr Oncol Rep 25:659–669. 10.1007/s11912-023-01396-3 36995533 10.1007/s11912-023-01396-3

[51] Smith GL, Banegas MP, Acquati C et al (2022) Navigating financial toxicity in patients with cancer: a multidisciplinary management approach. CA Cancer J Clin 72:437–453. 10.3322/caac.21730 35584404 10.3322/caac.21730 PMC12994614

[52] Tremblay D, Touati N, Usher S et al (2023) The challenge of optimizing supports for people living with and beyond cancer: creating proximity between cancer and non-profit community-based providers. Support Care Cancer 31:1–11. 10.1007/s00520-022-07569-3 10.1007/s00520-022-07569-3 PMC9831956 36625923

[53] Brennan L, Sheill G, O’Neill L et al (2022) Physical therapists in oncology settings: experiences in delivering cancer rehabilitation services, barriers to care, and service development needs. Phys Ther 102:1–8. 10.1093/ptj/pzab287 10.1093/ptj/pzab287 PMC8887570 35084029

[54] Sharma R, Molinares-Mejia D, Khanna A et al (2020) Training and practice patterns in cancer rehabilitation: a survey of physiatrists specializing in oncology care. PM R 12:180–185. 10.1002/pmrj.12196 31140751 10.1002/pmrj.12196 PMC7967832

[55] Rafn BS, Midtgaard J, Camp PG, Campbell KL (2020) Shared concern with current breast cancer rehabilitation services: a focus group study of survivors’ and professionals’ experiences and preferences for rehabilitation care delivery. BMJ Open 10:e037280. 10.1136/bmjopen-2020-037280 32723743 10.1136/bmjopen-2020-037280 PMC7389511

[56] Neris RR, Leite ACAB, Nascimento LC et al (2020) “What I was and what I am”: a qualitative study of survivors’ experience of urological cancer. Eur J Oncol Nurs 44:101692. 10.1016/j.ejon.2019.101692 31751850 10.1016/j.ejon.2019.101692

