# Supplementary material for: Exploring patients and caregivers needs and experiences in oncological physiotherapy: a call for collaborative care
Source: Support Care Cancer. 2024 Aug 19;32(9):594. doi: 10.1007/s00520-024-08782-y (PMC11330947; doi:10.1007/s00520-024-08782-y)
Supplement: Supplementary file 1 — Supplementary file1 (DOCX 29 KB) [file 520_2024_8782_MOESM1_ESM.docx]

| Theme | Subtheme | Quotes |
| --- | --- | --- |
| **Feeling cared for** | **Humanization** | **Vocation:**  *"The doctor said to my wife: 'Don't worry Pilar, I've heard you before that you claim an ultrasound, I've been looking at your file and no one comes to the hospital four weeks in a row if they don't have pain. We're going to do the ultrasound now" and that's when they told her she had."* (C_03)  **Empathy:**  *" A bit of empathy, dam it, we’re not infected by the plague."* (C_01)  *"Proximity is essential... Let them look you in the eye."* (P_04)  *"Here, of course, empathy does not exist, and it is always very important in the illness, above all."* (C_02)  **Ethics:**  *"It seems that it is cancer patients and families who have a special virus in which no one approaches, gets infected and everyone moves away."* (C_03)  *"I understand that they took less interest in my father than in my husband."* (C_04)  *"Protocol and the protocol go as far as here, and I'll get a little less so as not to go over the protocol."* (C_03)  *"But apart from that, perhaps, as you say, protocols that may exist, I think that many times it is a lack of time."* (C_07)  *"I understand that it has to be difficult because in the end with patients and limited time and resources and a lot of things and frustration."* (P_05)  **Communication:**  *"And I thought yes, yes, yes, they've explained everything to me super well. And suddenly, the first sentence he blurts out and says, "So have you been told that your penis is going to get shorter?" No."* (P_06)  *"The thing is that it's true that first of all men find it difficult to express our feelings."* (P_06)  *"I'm in a very vital moment; For me, these meetings and also with people I don't know at all, and I think you open up in a certain way, that you tell your experiences. All of this is vital to me."* (P_06)  *"They say no, as things go by, we count. But I need to know, uncertainty kills me."* (C_04) |
|  | **Counselling** | **Who offers:**  *"I am eternally grateful to the oncologist because she gave us her private phone, we called and wrote to her whenever we had any questions. She's lovely."* (C_07)  *"But they provided us with all the solutions at all times and advised us and told us at all times what the solutions were."* (C_01)  *"The surgeon who operated on me knew I was going to lose a lot more muscle and so on, and it didn't occur to him to tell me to go to a physio."* (P_03)  *"To me with my physio, with Marimar, very good, but she is the one who has explained things to me, because they don't give you information about anything."* (P_07)  *"Even Marta the other day informed me of things because she informs you, but I told Marta well, let's see, and I don't quite understand this, because if this problem removes the lymph nodes, there is no drainage of the lymphatic fluid. If you wear a sleeve that suits you, how the does it work? Because in the same way that it doesn't let you out, it doesn't let you in."* (P_01)  *"The oncologist was there for me and my wife at all times; I was her guardian angel."* (C_01)  *"But I developed lymphedema at the age of ten, and maybe if I had been told things from the beginning I shouldn't have done. What you're saying, about taking the weight sometime, well, no one has said anything to me, you just have to take care of this arm."* (P_07)  **Who doesn’t:**  *"My wife didn't eat either. Everything made him gag. At the Cancer Association. The nutritionist here is the first person who gave me some guidelines for eating."* (C_03)  *"Unfortunately, there are more and more people in this disease, and no, the services are not growing."* (C_02)  *"There came a time, for example, when I had to help him and not know how to manage mobility..."* (C_05)  *"She didn't get out of bed, and I saw her sink into bed and stay still and I had no idea about anything. He didn't want to move."* (C_04) |
|  | **Access** | *"I have to tell the rehabilitator: look at how my arm is. And then they say, well, they'll call you, they'll call you. It's just that in the end I have to go now because I know Mari, I'm going then I'm going particularly."* (P_02)  *"If you don't go to a rehab because they're treating you, you're being treated by an oncologist, shit. How do you get to the physio?"* (C_07)  *"We have paid for all the physiotherapy sessions."* (C_02)  **Resources:**  *"I have both shoulders necrotic from chemotherapy and in the social security they gave me some rehabilitation. I don't have a lymphatic system in my legs because they emptied me completely when I was 38 years old and I have to pay for my pressotherapy, I have to pay for my physios because no one helps me and with my daughter the only ones who have helped me a little is Aspanoa."* (C_02)  *"... The physio, those months he was with the foot, because it's an important budget. And I told him, thank goodness, that we have that possibility because not everyone can. So, what does it do? Don't walk or take opiates, which is what they gave us back then. And it was all done privately."* (C_07)  **Rural:**  *"Where I am, they either have to go to Calatayud or they have to come to Zaragoza, they have to die at the post. And if you don't have one, you have to make a living. It's not the same, they have to send you, you have to send derivative."* (P_07) |
|  | **Specialization** | *"It has to be a specialist, not all physiotherapists know what to do."* (P_04)  *"A girl from Granada came, a young woman who that same day didn't even know where the idea was going, she was very nice, she was super worried, but well, I have no idea. I had to be telling him what I had to do (....) well, look, now put the cuffs on and well, look, do this, this, put on such-and-such, and now you sell me. So of course, the first day he bandaged me terribly, he put his hand on me...!"* (P_01)  *"Well, but apart from that, they didn't foresee it. Not just because the girl doesn't have the experience, for whatever reason, they're basically telling you that it wasn't that important, or they don't give it to her. They don't give it to us."* (P_01)  *"I think we need a lot more people who specialize in lymphedema. Specifically in the field of oncology."* (P_07) |
| **The role of physiotherapy in the oncological process** | **Info: I didn't know that physiotherapy is good for this/has that potential** | **Preventive treatment:**  *"He endured more precisely because of the strength he had to exercise and eat well and such."* (C_07)  *"You can't leave yourself; you can't leave yourself, you can't, you can't. And you notice it. You notice improvement."* (C_08)  *"He endured more precisely because of the strength he had to exercise and eat well and such."* (C_07)  *"You can't leave yourself; you can't leave yourself, you can't, you can't. And you notice it. You notice improvement."* (C_08)  **Treatment of sequelae:**  *"The physio came and the first day my arm hurt and I told her: 'My arm hurts a lot, we can't leave it unmoved, and that is achieved.”* (P_03)  *"And he said to me, and this for the rest of your life, count on it."* (P_03)  *"That's why I learned about the importance of physiotherapy there, because he was the one who made me see the importance of physiotherapy in my life."* (P_03)  *"They do the lymphatic drainage, then they put the pressotherapy and the truth is that I am very good. I arrived with my arm and hand with a jug and well."* (P_03)  *"In the world I've been in with multiple disabilities, disability in schools is full of physios. Of course, apart from treating them, they also give them harnesses, devices, parts..."* (P_01)  *"In August, a kid was hit in a bike accident, and they took him to the reserve and they have pulled him through. In other words, he was left with trauma. Well, thanks to every day in the gym, every day, that the guy goes, he doesn't go anymore, and he doesn't wear a diaper. He's going, he's walking, he's no longer in a wheelchair."* (P_03)  **Changing myths:**  *"Physiotherapy is a new thing, an emerging thing, which is also now very, very fashionable and I think it's phenomenal."* (P_03)  *"Physical therapy is also teaching."* (P_03)  *"In life it would have occurred to me that a man can do a pelvic floor, but in life."* (P_06) |
|  | **Pillar in the teams** | *"The nurse at the Day Hospital..."They do some surveys and offer you things that clarify doubts when you have not lived with cancer, but they do not name you; me at least. They haven't appointed me physiotherapy."* (C_07)  *"Curious, well, curious to call it something. It's just that the times we've been in the hospital, from time to time the priest would come by, but the physio has never passed."* (C_07)  *"And the problem that I've also seen is that there's no information. It's that, for example, these therapies doctors should inform patients."* (C_03)  *"As Paco rightly says, he says we talk about the health world and we think of doctors, nurses. But a physiotherapist doesn't come to mind at first. To me at least, yes. So I think they should be integrated in such a way that they are part of it. So, it should be a priority.”* (P_01) |
|  | **Previous perceptions regarding physiotherapy** | **Negative emotions:**  *"He didn't want to go to the physio either because he was afraid that it would bother him with other things."* (C_01)  *"In the beginning, when they did it to me at the Servet, they gave me manual lymphatic drainage, they did just that. I have the experience there and it was bad (...) The nurses did it, and they hurt me! I remember that they put their arm around my arm sitting on a chair and my arm resting on it. I would go home with back pain that I couldn't have."* (P_07)  *"My wife just didn't want to because I don't know, ... She has also admitted that she was very embarrassed to undress."* (C_01)  **Positive emotions:**  *"He spent eight months telling me that it was nothing, that it was a sprain until the physio saw it."* (P_07)  *"So, opiates were a solution to take away the pain, but not to take away the problem that the physio later discovered, which is that he had some edema, some broken bones and such."* (C_07) |
